# The effect of acupuncture on gastrointestinal recovery after abdominal surgery: a narrative review from clinical trials

**DOI:** 10.1097/JS9.0000000000001641

**Published:** 2024-05-17

**Authors:** Qi Kong, Li-Ming Chen, Chu-Yu Liu, Wei Li, Pei-Hao Yin

**Affiliations:** a Department of General Surgery, Putuo Hospital, Shanghai University of Traditional Chinese Medicine, Shanghai, China; b Interventional Cancer Institute of Chinese Integrative Medicine, Shanghai University of Traditional Chinese Medicine, Shanghai, China; c Yueyang Hospital of Integrated Traditional Chinese and Western Medicine, Shanghai University of Traditional Chinese Medicine, Shanghai, China; d School of Acupuncture-Moxibustion and Tuina, Shanghai University of Traditional Chinese Medicine, Shanghai, China

**Keywords:** abdominal surgery, acupuncture, clinical research, enhanced postoperative recovery, gastrointestinal function

## Abstract

Abdominal surgery is a critical surgery, with more and more attention being paid to postoperative life quality and associated complications in recent years. Among these complications, postoperative gastrointestinal dysfunction is the most common complication of abdominal surgery. Acupuncture therapy is a treatment approach based on the Traditional Chinese Medicine theory, and its feasibility in aiding gastrointestinal recovery after abdominal surgery is supported by both Traditional Chinese Medicine theory and animal experiments. A lot of clinical research has been conducted to evaluate its efficacy, albeit with limitations, and at preliminary stages. Moreover, intervention timing, acupoint selection, and patient benefits should also be considered in clinical practices. This article summarizes the progress of clinical research on acupuncture therapy in gastrointestinal recovery after abdominal surgery and discusses related issues and operations, with the aim to provide new insights and prospects for the incorporation of acupuncture into the Enhanced Recovery After Surgery protocol.

## Introduction

Highlights

- The feasibility of acupuncture to aid gastrointestinal recovery after abdominal surgery is supported by Traditional Chinese Medicine (TCM) theory, animal experiments, and clinical trials.
- During the perioperative period, patients experience three types of stimuli: anesthesia, neurologic stimulation, and inflammatory response. Acupuncture effects on neurologic stimulation and inflammatory response.
- Timing of intervention, point selection, and patient benefit should also be considered in the clinical practice of postoperative abdominal acupuncture.

Abdominal surgery, involving laparoscopic or open procedures, presents a critical aspect of surgical practices^1^. With the advancement of surgical technologies, abdominal surgery has transitioned from an open operative style to a minimally invasive and laparoscopic style over the past two decades^2^. It usually takes a long time for patients after abdominal surgery to recover^3^. Postoperative life quality and associated complications of patients have been increasingly focused on in clinical practices. Postoperative gastrointestinal dysfunction (POGD) is a common complication of abdominal surgery, including postoperative ileus (POI)^4^ and postoperative nausea and vomiting (PONV)^5^. Not only does this complication exert a threat to patients’ health and life quality, but it also imposes a substantial economic burden on patients^6^. Enhanced Recovery After Surgery (ERAS), which is a multimodal, multidisciplinary, and evidence-based approach to patient care, has gained significant attention in recent years^7^. Initially implemented in colorectal surgery, ERAS has virtually presented improved outcomes in all major surgical specialties^8^. However, the current implementation effectiveness of ERAS has been compromised by factors such factors as lack of manpower, ineffective communication and collaboration, resistance to change, and patient-related issues^9^, as well as insufficient evidence-based support^8^.

Acupuncture, as a treatment method based on TCM, is considered to have therapeutic effects on gastrointestinal functions^10^. As a traditional therapy in TCM, acupuncture is regarded as a safe and effective way of treating POGD (such as POI)^11,12^. Therefore, some scholars have argued that acupuncture can help restore gastrointestinal functions among postoperative tumor patients and proposed to include it in the ERAS protocol^13^. This paper aims to summarize the progress of clinical research on acupuncture intervention in recovering gastrointestinal functions of patients after abdominal surgery. With related issues and procedures discussed, this paper provides a new perspective for including acupuncture in the ERAS protocol.

## Postoperative gastrointestinal complications and pathogenesis of abdominal surgery

### Postoperative gastrointestinal complications of abdominal surgery

POGD is the most common complication among patients after abdominal surgery, including POI and PONV, among others. Although POI can be viewed as one type of POGD, its definition remains unclear^14,15^. Therefore, normally, it is difficult to perform a rough classification of POGD. In 2018, to describe the clinical manifestations of gastrointestinal diseases more accurately, the American ERAS Society adopted a functional definition and scoring system on POGD as follows^16^: normal, postoperative gastrointestinal intolerance (POGI), and POGD. In this scoring system, “normal” patients may include patients with PONV. “POGI” patients may present no bowel movements or farting on top of PONV symptoms, with no use of nasogastric tubes required. “POGD” patients normally exhibit POI-like symptoms, including tympanic abdominal distention, absence of bowel movements, antiemetic-resistant nausea, and copious biliary vomiting. Approximately 10.3% of patients after laparotomy, laparoscopic cholecystectomy, and colectomy will develop a POI^17^, leading to longer hospital stays, increased costs, and higher readmission rates^17^. There could be multiple factors leading to POGD, with no definite conclusions drawn up to now.

### Pathogenesis

#### Use of anesthetic drugs

PONV is a typical symptom of POGD. A number of risk factors predispose patients to PONV, with postoperative use of opioids regarded as one of the most important risk factors^18^. Opioids have long been used as the most effective analgesics^19^. A study in the United States shows that up to 36.5% of all surgery use opioids^20^. Opioids, as the most commonly used drugs in surgery, present an undeniable impact on gastrointestinal functions, causing constipation^21^ and even leading to POI^4^. It is commonly believed that these gastrointestinal side effects can be mediated by opioid agonists bound with μ-receptors located in the enteric nervous system, increasing in nonpropulsive contractions, inhibited water and electrolyte excretion, and delayed gastrointestinal transport and constipation^22,23^. With the slowed gastrointestinal transport, opioids can also significantly alter the intestinal microbiota^24^, and even exacerbate existing gastrointestinal diseases^25^. Other than opioids, some studies have shown that volatile anesthetics are the main cause of early postoperative vomiting^26^, which is contradicted in some current studies^27,28^.

#### Surgical trauma and inflammation

Typically, the incidence of POGD is closely related to surgical trauma^29,30^. Neurogenic and inflammatory mechanisms of human bodies can influence their responses to surgical trauma^31^. Generally, surgical trauma affects the onset of POGD during the following two stages. The first stage is at the start of surgery, and primarily, surgical trauma at this stage can be neurologically mediated. Skin incisions during surgery can activate mechanoreceptors and nociceptors, inhibiting gastroenteric neural pathways and leading to gastroparesis^32^. In terms of intestines, on the one hand, skin incisions and laparotomy can induce inhibitory spinal reflexes and sympathetic reflexes or adrenergic inhibitory pathway activation, thereby temporarily inhibiting intestinal motility^33,34^. On the other hand, intestinal manipulation will trigger additional high-threshold supraspinal pathways, with specific hypothalamic and pontine neurons (such as nuclei of the solitary tract, periventricular nuclei, and supraoptic nuclei of the hypothalamus) activated. In this way, corticotropin-releasing factors can lead to acute POI^33^. Some scholars argue that, with intestinal neural reflex pathways inhibited, sympathetic pathways responding to surgical trauma would be activated, thus improving gastrointestinal functions that are inhibited^35^. Besides those pathways mentioned above, intense visceral afferent stimuli caused by surgery can also induce other nonadrenergic neuron-mediated pathways. For example, animal experiments verified that during cecum removal after small intestine manipulation, additional vagus nerve-mediated inhibitory nonadrenergic and noncholinergic pathways could be activated, participating in the relaxation of gastric fundus among rats after abdominal surgery^36^. Endogenous gases also seem to play a mixed role. Research showed that the additional inhibitory effect of mechanical stimulation during abdominal surgery is caused by the enhanced release of nitric oxide from constitutive nitric oxide synthase^37^. In addition, mice treated with carbon monoxide present improved POI^38^. Furthermore, it seems that hydrogen sulfide has a partial inhibitory effect on POI^39^, with a complex mechanism and various influencing factors. Some endogenous gases can be used as therapeutic targets to improve the POGD of patients after abdominal surgery. However, their mechanisms need to be further investigated.

The second stage is during the postoperative period, with surgical trauma primarily mediated through inflammation. In abdominal surgery, there are gastrointestinal inflammatory responses to intestinal manipulation or surgical trauma. Traumatic inflammation can cause local releases of 5-hydroxytryptamine and other mediators and affect the signaling of exogenous afferent fibers, thus eventually leading to PONV^40^. Furthermore, a series of animal experiments and human specimen studies showed that there is an increase in leukocyte infiltration during postintestinal handling^41,42^. Intestinal manipulation can induce mast cell degranulation^43^ and activation^44,45^, with resident macrophages activated by mast cell-derived mediators or luminal antigens^46,47^. Under such a circumstance, cytokines and chemokines are generated, directly impairing the contractility of intestinal smooth muscle cells through the releases of nitric oxide^48^ and prostaglandins^49^, subsequently inhibiting intestinal contractile activities and leading to intestinal dysfunctions. The role played by vagus nerves in this process should be noted. Vagus nerves play a critical role in regulating metabolic homeostasis, with efferent vagal cholinergic signals controlling immune functions and pro-inflammatory responses via inflammatory reflexes^50^. Activated vagus nerves can inhibit inflammation induced by intestinal manipulation through the α7-nicotinic acetylcholine receptor-mediated JAK2/STAT3 signaling pathway^51^. Meanwhile, vagus nerve stimulation leads to significantly decreased interleukin (IL)-6 and IL-8 among patients undergoing gastrointestinal surgery^52^. This indicates that activated vagus nerves could influence the vagus nerve stimulation efficacy, which can further affect macrophages through intestinal neurons. Studies have confirmed that abdominal surgery can activate M1 macrophages and improve the expressions of proinflammatory cytokines, resulting in inflammation of gastrointestinal muscle plexuses and delayed gastric emptying^53^. Central vagal stimulation can halt increases in M1 macrophages and proinflammatory cytokines, reduce neutrophil infiltration and suppress POGI 6 h after abdominal surgery. The possible reason is that there are synaptic interactions between vagal nerve endings and enteric neurons around muscularis macrophages^52^.

## Existing clinical research of acupuncture intervention in postoperative gastrointestinal function recovery after abdominal surgery

In recent years, clinical research on acupuncture intervention in postoperative gastrointestinal function recovery after abdominal surgery has received wide attention. A meta-analysis of 15 experiments involving 965 patients^54^ shows that electroacupuncture (or transcutaneous acupoint electrical stimulation) is a safe and effective intervention method for POI after abdominal surgery (including laparoscopic surgery). On average, the time to first flatus and time to first defecation can be reduced by more than 10 h, with 1.19 days of hospitalization saved. However, its efficacy in alleviating postoperative pain remains uncertain^55^. Therefore, a consensus among experts may be required to clarify its treatment protocol. Other meta-analyses have presented similar conclusions^11,56^. High-quality clinical research on acupuncture intervention in postoperative gastrointestinal function recovery after abdominal surgery during recent years is summarized in this paper, with the aim of providing a basis for future studies.

### POI

POI is one of the major complications after abdominal surgery, especially after colorectal cancer (CRC) surgery. Recent high-quality clinical research^57–59^ has confirmed the superior efficacy and safety of acupuncture intervention in POI after CRC surgery, with electroacupuncture considered a more effective treatment method than sham acupuncture^58^. Compared to other acupoints, Tianshu (ST25) and Zusanli (ST36) can significantly enhance the recovery of intestinal functions among CRC patients after elective laparoscopic surgery^57^. The times to first flatus and first defecation are the most commonly used primary measures of acupuncture outcomes, with an acupoint of ST36 required and most interventions performed after the operation^11^. However, research shows that acupuncture cannot be viewed as a significantly effective way of preventing long-term POI^60^. Also, some clinical research further confirmed through animal experiments^61,62^ that electroacupuncture can alleviate postoperative intestinal inflammation by activating α7-nicotinic acetylcholine receptor-mediated JAK2/STAT3 signaling pathway through vagus nerves, thereby improving POI. In addition, acupuncture has been used in the treatment of POI relating to gastric cancer^63,64^ and gynecological surgery^65^. However, there is a lack of related clinical research and mechanism studies.

### PONV

PONV is the most common complication of abdominal surgery. For example, in laparoscopic surgery, under the influences of surgical stimulation, CO_2_ pneumoperitoneum, and anesthetic drugs, PONV is an almost inevitable symptom^5^. In clinical practices, PONV is mainly treated with drug therapy. However, continuous drug therapy often leads to various adverse reactions. Acupuncture intervention after laparoscopic surgery is the most common subject studied among all relevant research using the acupoint of Neiguan (PC6)^66^. Research shows that acupuncture at PC6 can alleviate the symptoms of nausea, with a possible mechanism related to the coupled regulation between the cerebellum and insula, which is a specific neurobiological basis^67^. A wide range of studies has been conducted on acupuncture intervention in PONV, with most of them focusing on PONV after laparoscopy, including laparoscopic CRC resection^68^, sleeve gastrectomy^69^, and gynecological surgery^70^. Moreover, its effectiveness at different surgery stages has received wide attention^71^. In addition to laparoscopic surgery, attention has been paid to open surgery too^72^. Overall, almost all acupuncture therapies have the potential to alleviate PONV, which can be treated through the combined effect of acupuncture and drugs^73^. Both acupoint stimulation and drugs can present considerable efficacy, with acupuncture treatment presenting a superior performance^74^.

## Acupoint selection and mechanism of acupuncture in promoting postoperative gastrointestinal function recovery after abdominal surgery

In TCM, POGD belongs to the disease category of “intestinal bloating,” “abdominal pain,” “vomiting,” and “constipation,” involving such organs as the spleen, stomach, large intestine, and small intestine^75^. TCM theory holds that surgery patients suffer from an “injury of sharp instruments.” In surgery, external pathogenic factors are dispersed, with normal tissues being harmed. Therefore, vital energy and blood are damaged and depleted, resulting in disrupted circulation of vital energy. All these dysfunctions can lead to disharmony of the spleen and stomach, whose functions are gradually weakened, thus resulting in gastrointestinal disorders. This indicates that acupuncture intervention in POGD has a solid theoretical foundation of TCM. A brief description of the TCM terminology in this chapter is provided in Supplementary Document 1, Supplemental Digital Content 1, http://links.lww.com/JS9/C614.

There are already rules for selecting acupoint for promoting postoperative gastrointestinal function recovery after abdominal surgery. For example, ST36 is the main acupoint selected for postoperative gastrointestinal function recovery after CRC surgery with the combination of Shangjuxu (ST37), PC6, Sanyinjiao (SP6), and other acupoints^75^. ST36, PC6, and SP6 are the main acupoints selected for gastrointestinal function recovery after gastric cancer surgery, with the most trusted combinations of ST36-PC6 and PC6-SP6-ST36^76^. One research on common POGD showed that ST36, ST37, PC6, SP6, and Tianshu (ST25) are acupoints most frequently used, with a possible core prescription of ST36-PC6-ST37-Xiajuxu (ST39)^77^. There is evidence that ST36 and PC6 are important acupoints for postoperative gastrointestinal function recovery after abdominal surgery, which is basically consistent with the results of the meta-analyses previously mentioned. The *Poem of Four Essential Acupoints* (四总穴歌) presents the phrase “The belly and stomach retain Sanli (ST36),” which was first seen in *Qiankun Shengyi* (乾坤生意)^78^. The use of ST36 in TCM intervention of gastrointestinal tissues has a theoretical basis. Research has shown that electroacupuncture at ST36 alone can lead to a quick recovery of gastrointestinal motility after colorectal surgery^79^. POGD after abdominal surgery is closely related to neurogenic changes and inflammation, which are two primary aspects of acupuncture at ST36 regulating human bodies. For instance, research has shown that electroacupuncture stimulation at ST36 can improve POGD in a pancreatitis model by regulating the enteric nervous system, especially acetylcholine neurons^80^. Furthermore, electroacupuncture at ST36 can improve neurotransmitter loss in diabetic gastroparesis by increasing the levels of inhibitory neurotransmitter nitric oxide synthase and excitatory neurotransmitter choline acetyltransferase in the gastric antrum, partially restoring damage to general neurons^81^.

A literature review of 69 studies shows that 27 studies have investigated the anti-inflammatory effect of ST36 acupuncture on the digestive system^82^. For example, in a POI model^62^, electroacupuncture at ST36 reduced concentrations of serum tumor necrosis factor-α and IL-6 and intestinal myeloperoxidase activity, which was measured to monitor macrophage and neutrophil infiltration. This finding indicates that electroacupuncture can inhibit local immune responses. It shows that ST36 electroacupuncture can effectively relieve systemic inflammation by inhibiting local intestinal inflammatory responses, thereby improving gastrointestinal transport. In addition, electroacupuncture at ST36 can reduce levels of tumor necrosis factor-α and vascular endothelial growth factor in adhesion tissue homogenate 7 days after surgery^83^, indicating that it could reduce postoperative local inflammatory responses, weaken angiogenesis, and decrease adhesion formation by activating cholinergic anti-inflammatory mechanisms.

PC6 is a collateral acupoint of the Jueyin Pericardium Meridian, communicating with the Shaoyang Sanjiao Meridian of hand, bridging exterior and interior meridians, and facilitating the Qi mechanism of the Sanjiao Meridian^84^. In TCM theory, it is considered that PC6 has an antiemetic effect. In modern medicine, it is normally believed that PC6 can improve PONV through the regulation mechanisms of gastrointestinal motility and neurotransmitters. Studies have indicated that electroacupuncture at PC6 and ST36 can enhance intestinal transit and gastric emptying in rats with POI, with the possible mediating effects of autonomic nerves and cytokines^85^. Moreover, transcutaneous electrical nerve stimulation at ST36 and PC6 can reduce the levels of vasoactive intestinal peptides, gastrin, and IL-6 in the plasma of patients with systemic sclerosis and improve gastric myoelectrical activities and the balance of sympathetic and vagal nerves^86^. In addition, both acupuncture and transcutaneous electrical acupoint stimulation can reduce gastrin levels among patients after gynecological surgery^87,88^. In summary, ST36 is the acupoint mostly studied in modern medicine, with significantly fewer studies focusing on the commonly used acupoint of PC6. Whether a combination of acupuncture at different acupoints would be more effective, as well as mechanisms of acupuncture at single acupoints and combined acupoints, need to be further explored. This study of ST36 has provided a reference and evidence for future clinical and animal experimental research on acupuncture therapy.

## Issues of acupuncture in promoting postoperative gastrointestinal function recovery after abdominal surgery

### Issues in existing clinical research

The application of acupuncture in ERAS has been a hot research topic in recent years^89^. However, in these clinical studies, there are significant issues that cannot be overlooked. First, most acupuncture clinical studies, including studies on acupuncture intervention in postoperative gastrointestinal function recovery after abdominal surgery, have common problems such as low-quality reports of acupuncture randomized controlled trials (RCTs), as well as numerous influencing factors and methodological challenges of acupuncture RCT efficacy^90^. A quality assessment of Chinese and English acupuncture RCT reports based on the Consolidated Standards of Reporting Trials statement and Standards for Reporting Interventions in Controlled Trials of Acupuncture checklist shows that the number of English reports is greater than that of Chinese reports on most items^91^. Therefore, this paper intends to cite high-quality English literature of relevant clinical research, considering that these studies could be more representative and credible. In addition, compared to drug interventions, acupuncture therapy relying on specialist expertise is usually complex and multifaceted^92^. Therefore, qualified professionals and even necessary quality control of clinical research efficacy through the monitoring of acupuncturists’ work experiences are generally required in acupuncture research. Furthermore it is noteworthy that the aims of acupuncture clinical research, the clinical problems to be solved, and the perspectives of researchers are closely connected^93,94^. Unlike conventional chronic diseases, postabdominal surgery complications are often acute, which is one of the primary reasons for extended hospital stays. This is an advantage of acupuncture intervention, which is typically easy to follow up in the short term. However, less research has focused on the long-term effects of acupuncture intervention on the gastrointestinal function recovery of postabdominal surgery patients. Some existing research has shown negative results on the effects of acupuncture intervention^62^. Nevertheless, to explore the role of acupuncture intervention, it may be necessary to extend the follow-up duration of patients’ hospital stays, even tracking the onset of other related diseases.

**Figure 1:**
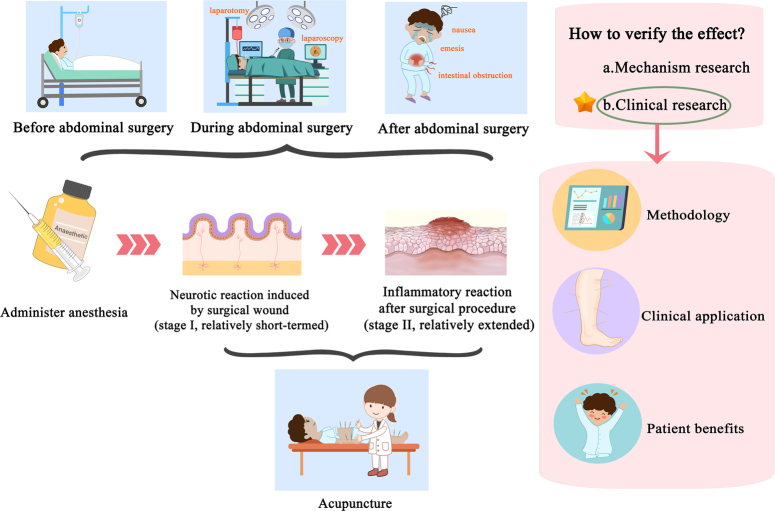
The use of acupuncture in abdominal surgery. During the perioperative period, patients experience three types of stimuli: anesthesia, neurologic stimulation, and inflammatory response. Mechanistically, acupuncture can affect neurologic stimulation and inflammatory response. The mechanism of acupuncture has been relatively well investigated. We advocate the validation of the actual effects of acupuncture on various types of injuries on abdominal surgery on the basis of ERAS. Optimizing trials by improving trial methodology, clinical operations, and focusing on patient benefit will result in more adequate clinical evidence. ERAS, Enhanced Recovery After Surgery.

In addition, existing acupuncture clinical research has methodological issues. Generally, only pilot trials with small sample sizes were performed in clinical research on postoperative gastrointestinal function recovery after abdominal surgery. Recent meta-analyses summarizing CRC-related clinical studies^11^ show that only one study involved has a total sample size exceeding 100 patients^59^. Sample size plays a decisive role in the credibility of research conclusions. Therefore, sufficient samples should be applied in relevant clinical research. Most acupuncture clinical research is single-centered, and there are few high-quality multicenter RCT studies^91^. This further undermines the credibility of research results. Moreover, most acupuncture RCT studies focus on Asian populations. However, to incorporate acupuncture into the ERAS protocol, not only is multicenter clinical research required, but studies should also expand their scopes to various populations (Fig. 1).

### Possible issues in the clinical application of acupuncture

#### Timing of intervention

The timing of intervention is a crucial factor in acupuncture treatment during the perioperative period. Most existing studies have focused on postoperative interventions^11^. However, the timing of intervention remains a matter of concern^71^. For instance, some research on laparotomy shows that preoperative acupuncture is more effective than intraoperative and postoperative acupuncture^95^. On the contrary, one study on laparoscopic cholecystectomy concludes that postoperative intervention is more effective than preoperative intervention^96^. It is worth noting that some scholars argue that the chemoreceptor trigger zone of the vomiting center can sense the release of a large amount of body fluid chemistry. Therefore, acupuncture treatment before surgery should be recommended^97^. However, from a practical point of view, it is often challenging to implement preoperative treatments, and with unclear symptoms, it is difficult to perform targeting acupuncture based on the specific conditions of patients. It takes different amounts of time for different parts of the gastrointestinal tract to restore peristalsis; that is, 6 h for the small intestine and 12/24 h for the colon. This affects the timing of postoperative acupuncture. Most current studies have focused on postoperative interventions. Therefore, research on preoperative and intraoperative interventions with more precise timing and a more comprehensive plan should be carried out.

Besides the intervention timing, DeQi may need to be considered in the intraoperative interventions due to the patients’ anesthesia states. DeQi, which is a specific sensation induced with stimulated acupuncture points, is considered a crucial factor affecting the therapeutic effect of acupuncture^98^. In clinical practices, DeQi, which patients usually report, can be primarily divided into two categories. One involves pain, prickling, sourness, heaviness, fullness, and numbness, while the other includes warmth, cold, and dullness^99^. However, in some studies, especially those children patient-involved studies^100,101^, acupuncture interventions were often performed after anesthesia induction, which may result in failing reporting of patients on their DeQi sensations. In addition, nerve fibers in the skins and muscles are often stimulated during acupuncture therapy^102^. Therefore, whether muscle relaxants commonly used in surgical anesthetics will affect the efficacy of intraoperative acupuncture (even though the acupuncture may be performed before anesthesia induction) needs to be considered.

#### Acupoint selection issues

In clinical research, to evaluate the maximum therapeutic effects of specific acupuncture techniques or protocols, highly standardized methods were used in intervention management and quality control^92^. Methods commonly used in current clinical research can be generally divided into two types, one of which is standardized acupoint prescription. For instance, in order to validate the efficacy of ST36, Yang *et al.* ^57^ used a single acupoint selection method in assessing high-quality clinical research. This method can fully evaluate the effectiveness of corresponding acupoints and facilitate subsequent research on the mechanism of acupuncture. However, it was primarily used in explanatory clinical research. Furthermore, it is worth noting that this method could waste resources. Therefore, from the point of view of benefit weighing, research should be conducted on specific acupoints, such as ST36 (POI) and PC6 (PONV).

Semi-standardized acupoint selection is the second common method type for selecting acupoints. As suggested in the Standards for Reporting Interventions in Controlled Trials of Acupuncture checklist^103^ for the description of acupuncture details, for treatment protocols that involve partially individualized prescriptions, it is necessary to list all required or optional acupoints in the prescriptions and describe the acupoints used in each visit with a conclusion of acupoints used on a certain basis. For example, acupuncture at six acupoints (five fixed acupoints with one of three optional acupoints) was administered based on patients’ syndrome diagnoses and specific acupoint matching principles^104^ in a study. It shows that different patients could be treated with different acupuncture prescriptions. This is normal in clinical practices and more suitable for pragmatic clinical research. However, acupuncture interventions are usually conducted before, during or immediately after abdominal surgery. These are predominantly preventive treatments because, in clinical practices, it is difficult for acupuncturists to treat patients with chief complaints immediately. Under such circumstances, patients’ postoperative life quality and subjective experiences will be negatively affected, which contradicts the ERAS and the principle of patient benefits. Selecting optional acupoints based on patients’ syndromes to provide personalized treatments is almost impossible with the specificity of surgery. Obviously, acupuncture interventions for abdominal surgery are significantly different from normal acupuncture interventions for chronic diseases. In addition, acupoint selection for intraoperative interventions needs to be considered. Proximal acupoint selection is an acupoint selection method based on the TCM theory. That is, acupoint prescriptions are made based on the therapeutic effects of locations and adjacent areas of acupoints selected^105^. Clearly, this is not practicable in open abdominal surgery. It could be feasible in laparoscopic surgery. However, more manipulation is still needed to determine whether acupuncture manipulation threatens surgical asepsis (Table 1).

**Table 1:** Potential problems of acupuncture in clinical application.

Common problems in clinical applications	Advantages and applicability	Shortcomings and issues
Intervention timing		
Preoperative intervention	More optional sites for preoperative acupuncture	Preoperative acupuncture can not improve the specific symptoms of patients, and compliance may be poor
Intraoperative intervention	Intraoperative intervention may reduce pain for patients, increase compliance, and can also assist in pain relief, potentially reducing the amount of anesthesia used	Acupuncture during surgery is difficult to ensure DeQi, anesthesia, and muscle relaxants may affect the effectiveness. The intervention may also affect the surgeon’s operation
Postoperative intervention	Able to treat patients based on their specific symptoms	Patients may not receive immediate treatment, and their compliance may be lower due to postoperative discomfort and emotional issues
Acupuncture prescription		
Standardized acupoint selection	Clear treatment prescription with strong repeatability	Commonly used in clinical trials, with poor clinical practicality
Semi-standardized acupoint selection	Specific treatment can be performed based on patients’ symptoms	The treatment prescription is not fixed and cannot stabilize the effect. Experts’ opinions are needed to clarify the semi-standardized acupuncture prescription

#### Ensuring patient benefits in acupuncture

Although acupuncture is considered a treatment method with high safety and minimal side effects, adverse reactions associated with acupuncture should not be overlooked. Approximately 7–11% of all patients undergoing acupuncture therapy have reported mild and transient adverse reactions, such as pain at the acupuncture locations^106^ and bleeding^107^. Also, fear of acupuncture due to pain or other reasons is a common patient experience^108,109^, with pain associated with acupuncture being almost inevitable. Some researchers argue that bleeding and pain in acupuncture are therapeutic manifestations and cannot be considered adverse reactions and that anticoagulants will not increase the risks of serious bleeding after acupuncture^110^. Under these circumstances, acupuncture practitioners should be obliged to inform patients to ensure their benefits. Patients during preoperative interventions may be less willing to accept acupuncture treatments due to their less manifested discomfort, while postoperative patients could be more willing to accept the treatments because they experience more physical discomfort. Therefore, how subjective experiences and emotional neuroregulation of patients influence the therapeutic effects of acupuncture throughout their treatment processes should be thoroughly investigated. Furthermore, it is noteworthy that currently, there are no effective interventions for certain postoperative symptoms. With POI taken as an example, enemas and laxatives can only provide temporary relief to POI symptoms while stimulating gastrointestinal tracts. Corresponding medications, such as the mast cell stabilizer Ketotifen and the 5HT-4 receptor agonist prucalopride, are still in the research and exploration phase^111^. In terms of symptoms for which no clinical medications are currently available, patients should be fully informed of the situation to ensure that they receive comprehensive information and are involved in the diagnostic and therapeutic processes. Based on this, patients can choose to undergo complementary and alternative therapies such as acupuncture to ensure their benefits, thus achieving better experiences of surgery.

#### Strengths and weaknesses

This review provides the first overview of the benefits and challenges of acupuncture in abdominal postoperative recovery. This overview is based on detailed research and practices, making it practically informative. To make it easy to understand, we have not focused on the reproducibility of the evidence synthesis approach in this article. Acupuncture is a treatment method based on traditional culture. In this review, we have focused more on its physical stimulation properties and ignored its cultural content to ensure that it is philosophically reliable.

## Ethical approval

Not applicable.

## Consent

Not applicable.

## Sources of funding

This study was sponsored by the One Hundred Talents Project of Putuo Hospital, Shanghai University of Traditional Chinese Medicine (2022-RCLH-03) and Shanghai Western Regional TCM Consortium and Shanghai Hospital of Traditional Chinese Medicine Branch 2022 “Future Program” TCM Inheritance Development Project (XBYLT-WLJH-2023-022).

## Author contribution

*P.-H.Y. and W.L.: contributed to the original idea. Q.K. and L.-M.C.: completed the first draft. Q.K.: completed the figure and table. L.-M.C. and P.-H.Y.: revised the manuscript. C-.Y.L.: translated and proofread. P.-H.Y. and W.L.: provided funding.

## Conflicts of interest disclosure

The authors declare no conflicts of interest.

## Research registration unique identifying number (UIN)

Not applicable.

## Guarantor

Pei-Hao Yin is the guarantor of this manuscript.

## Data availability statement

Data statements are not applicable to this manuscript.

## Provenance and peer review

This manuscript is uninvited.

## Presentation

Not applicable.

## Supplementary Material
