# Supplementary material for: The effect of acupuncture on gastrointestinal recovery after abdominal surgery: a narrative review from clinical trials
Source: Int J Surg. 2024 May 17;110(9):5713–21. doi: 10.1097/JS9.0000000000001641 (PMC11392097; doi:10.1097/JS9.0000000000001641)
Supplement: Supplementary file 1 [file js9-110-5713-s001.docx]

Description of acupoints and TCM terminology

The content of this description mainly references *WHO standard acupuncture point locations (Western Pacific Region)*.

**Tianshu 天枢 “Celestial Pivot” (ST25)**

On the upper abdomen, 2 B-cun lateral to the center of the umblilicus. Front Mu of the large intestine. It regulates the intestines, the spleen and stomach

**Zusanli 足三里 “Leg Three Li” (ST36)**

On the anterior of the leg, on the line connecting ST35 (DuBi) with ST41 (JieXi), 3 B-cun inferior to ST35. It is located on the tibialis anterior muscle. It harmonizes the stomach, fortifies the Spleen and resolves dampness. Point of the Sea of Water and Grain.

**Shangjuxu 上巨虚 “ Upper Great Hollow” (ST37)**

On the anterior of the leg, on the line connecting ST35 (DuBi) with ST41 (JieXi), 6 B-cun inferior to ST35. It is located on the tibialis anterior muscle. It regulates the intestines and transforms stagnation, and clears damp heat and alleviates diarrhea and dysenteric disorder.

**Xiajuxu 下巨虚 “ Lower Great Hollow” (ST39)**

On the anterior of the leg, on the line connecting ST35 (DuBi) with ST41 (JieXi), 9 B-cun inferior to ST35. It is located on the tibialis anterior muscle, at the same level as GB35 and GB36.

**Sanyinjiao 三阴交 “Three Yin Intersection” (SP6)**

On the tibial aspect of the leg, posterior to the medial border of the tibia, 3 B-cun superior to the prominence of the medial malleolus. 1 B-cun superior to KI8. Meeting Point of the Spleen, Liver, and Kidney meridian. It tonifies the spleen and stomach, resolves dampness and harmonizes the Liver and tonifies the Kidneys.

**Neiguan 内关 “Inner Pass” (PC6)**

On the anterior aspect of the forearm, between the tendons of the palmaris longus and the flexor carpi radialis, 2 B-cun proximal to the palmar wrist crease. Luo (connecting) point. It unbinds the chest, regulates the Heart and calms the spirit.

**Jueyin Pericardium Meridian 厥阴心包经**

Jueyin pericardium meridian of hand originates from the chest and pertains to the pericardium. It pass through the diaphragm and successively links with the upper, middle and lower energizer.

**Shaoyang Sanjiao Meridian 少阳三焦经**

Shaoyang Sanjiao Meridian starts at the ulnar aspect of the tip of the ring finger and goes between the fourth and fifth metacarpal bones along the dorsum of the hand. It moves up the posterior aspect of the forearm between the radius and the ulna and between the Large and Small Intestine meridians.

**Poem of Four Essential Acupoints 四总穴歌**

The belly and stomach retains Sanli (ST36) 肚腹三里留

The waist and back seeks Weizhong (BL40) 腰背委中求

The head and neck find Lieque (LU7) 头项寻列缺

The face and mouth collects Hegu (LI4) 面口合谷收
